# Compact bandpass pixelated microwave filters with short-circuited stubs via inverse design

**DOI:** 10.1038/s41598-025-10666-y

**Published:** 2025-07-15

**Authors:** Miguel A. Gomez, Wei Jia, Steve Blair, Berardi Sensale-Rodriguez

**Affiliations:** Department of Electrical and Computer Engineering, The University of Utah, Salt Lake City, UT 84112 USA

**Keywords:** Bandpass filters, Inverse design, Laser ablation, Microwave engineering, Notch filters, Pixelated metasurfaces, RF filter design, Shorted stub filters, Electrical and electronic engineering, Applied physics

## Abstract

Pixelated RF metasurfaces are poised to revolutionize electromagnetic component design by enabling compact, versatile, high-performance solutions. Building upon our prior work in random metasurface-based filters and inverse design methods, we propose pixelated notch filters by integrating shorted stubs within a top ground plane. Using a combination of established optimization techniques, including direct binary search optimization, genetic algorithms, and a randomization mutation algorithm, we synthesize filters enhanced by parallel short-ended feed schemes, which are shown to improve stopband response. Design iterations are automated via Python scripting, commercial full-wave simulations, and Visual Basic within the electromagnetic solver, overcoming initial seeding challenges and enabling innovative pattern-generation techniques. For implementation, laser ablation is employed to precisely remove copper on PCBs, streamlining fabrication on Rogers Kappa 438 substrates. Preliminary results demonstrate the ability of the approach to reach target insertion loss levels with compact geometries, advancing pixelated metasurface-based filter design with enhanced tunability and overall performance.

## Introduction

The development of pixelated RF metasurfaces has opened new avenues for the design of compact, high-performance electromagnetic components. Metasurfaces have been widely explored for applications in guided electromagnetic-wave manipulation, slow-wave structures, and frequency-selective surfaces^1^. Prior research introduced random metasurface-based filters, demonstrating their capability to outperform conventional microstrip filter designs in terms of size and frequency response^2^. Their utility is not limited to filters as they have been shown to provide a reasonable basis for performing algorithmic optimization of MIMO antenna systems with machine learning, significantly accelerating their design^3,4^. In addition to filters, these planar designs have also been applied to low-loss, broadband output matching networks for use in power amplifier matching^5,6^. Furthermore, related inverse design methodologies have been successfully employed in terahertz band-pass filters, diffractive optics, etc., optimizing their performance through advanced computational techniques^7–13^. Optimization techniques like Genetic Algorithms (GA) have also been investigated to further refine metasurface performance and tailor responses for specific applications^12,14–17^.

This work builds upon the foundational methodologies introduced by some of us in our previous work^2^ by extending their application to spectral notch filters and incorporating shorted elements within a coplanar waveguide structure. This investigation focuses not on improving optimization algorithms but on enhancing filter performance through novel structural integration of shorted stubs with pixelated metasurfaces. Shorted elements in filter design, as well as parallel short-ended feed schemes, have been shown to provide stopband-enhanced ultra-wideband (UWB) passbands using ring resonators^18^. In addition to these UWB passband filters, shorted filter designs have shown good performance and realizability^19^. A unique contribution of this work is the integration of shorted stubs to act as capacitive or inductive elements alongside the pixelated metasurface designs, inspired by their established use in traditional filter topologies to enhance performance and tunability of the notch^20^.

**Fig. 1:**
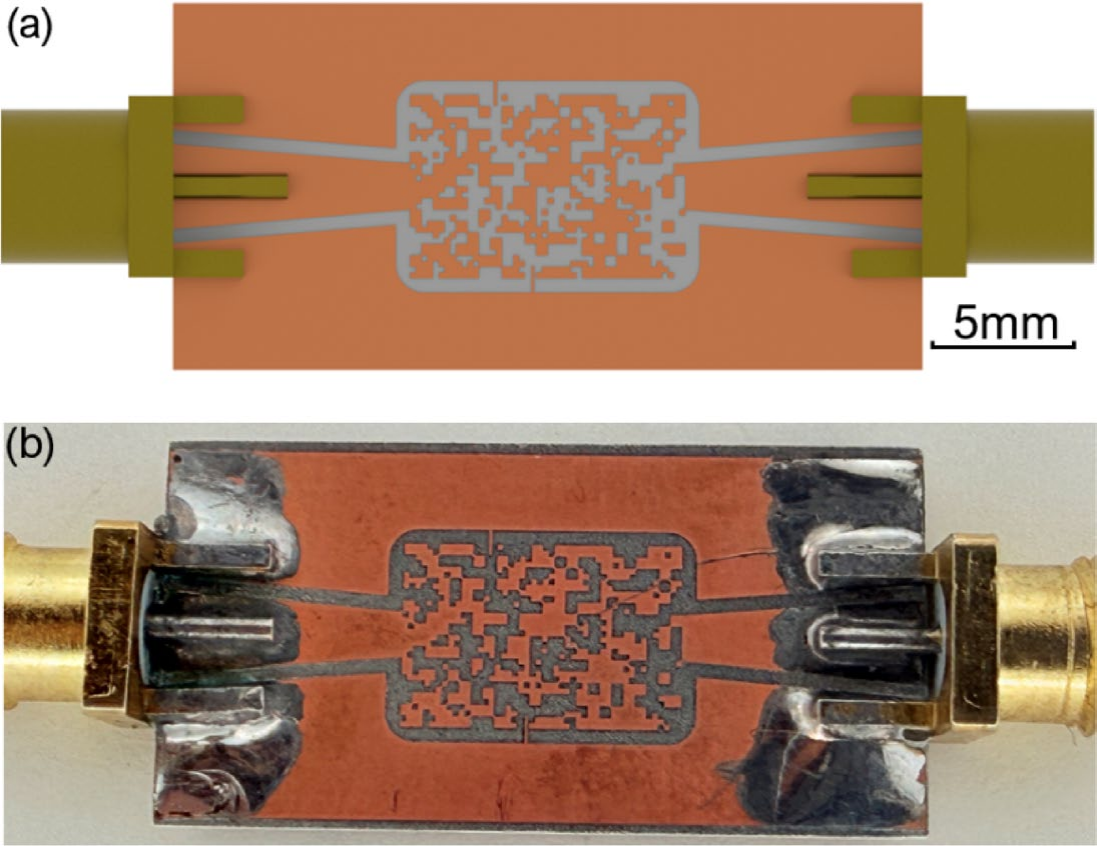
(**a**) Proposed pixelated microwave notch filter schematic. (**b**) Photograph of the fabricated filter.

The design and optimization processes are facilitated through CST Studio for electromagnetic simulations, Python for computational tasks, and Visual Basic (VBA) for automated design updating and iteration. To validate the proposed methodologies, filters are fabricated on a Rogers substrate, demonstrating efficient implementation with low insertion loss and more compact geometries than conventional approaches^21^. The schematic of the inverse-designed pixelated microwave notch filter is shown in Fig. 1(a), with the fabricated design presented in Fig. 1(b).

### Design and fabrication

The design uses a dielectric substrate from Rogers Corp., specifically Kappa 438 laminate^22^. This was chosen in part because of its low loss compared to standard FR-4 dielectric boards that we employed in our previous work^2^. The software used to simulate the electromagnetic response of the filter was CST Studio. This software can run simulations in parallel and supports various optimization algorithms such as GA. GA is a class of optimization techniques inspired by the principles of natural evolution and genetic variation^17^. In the context of designing electromagnetic (EM) filters, particularly when employing random pixelated designs, these algorithms can offer a robust means to explore vast and complex design spaces where conventional gradient-based methods may falter. The use of these algorithms has been shown with successful results in^23–25^. It is important to note that this work does not aim to improve these established optimization algorithms but applies them as tools to achieve the novel design approach. A key innovation of this work lies in integrating shorted stubs with pixelated metasurfaces to enhance filter performance, extending the pixelated metasurface concept that we earlier discussed in^2^ from a dual band filtering response to a more complex notch filter response. We employ these computational techniques to effectively explore the design space before introducing the stubs, not to advance algorithmic efficiency itself.

**Fig. 2:**
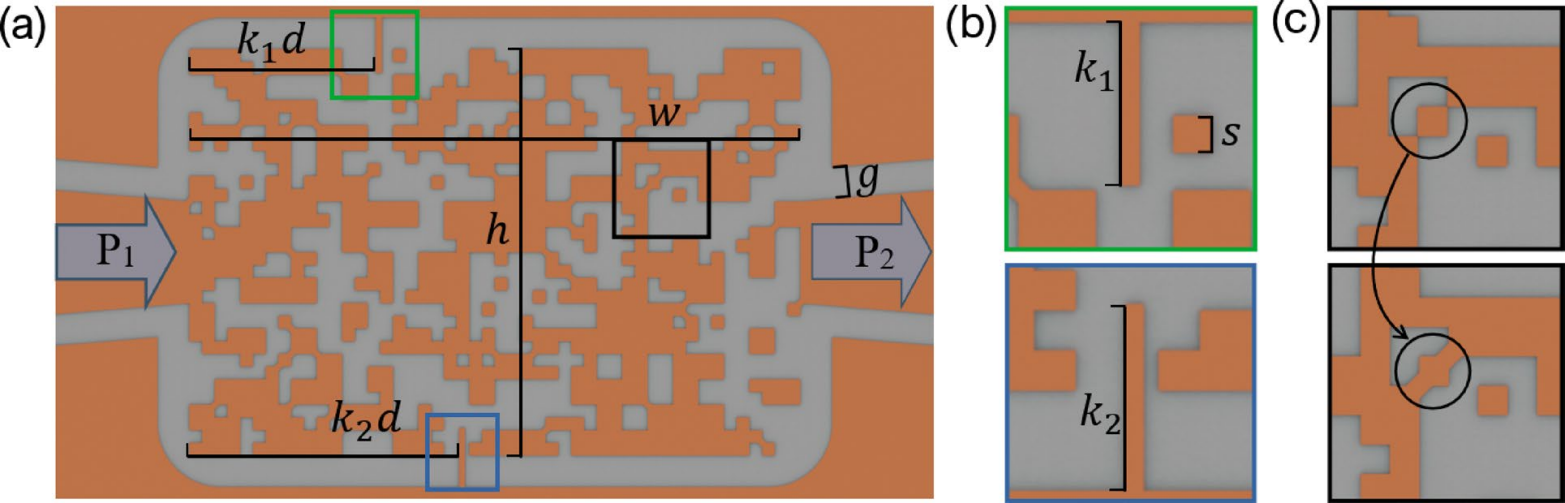
(**a**) Dimensions of the designed filters. (**b**) Included stubs at the top and bottom regions. (**c**) Added patches to ensure pixel connections.

A design region with specified height and width is defined for the simulation and shown in Fig. 2 whose dimensions are detailed in Table 1. These dimensions form the area in which the pixelated pattern is placed. In addition to the design region, a top ground plane is defined near the pixelated domain, which can provide a closer path to ground than what is possible through a classical microstrip configuration. This conductor boundary is included to create a geometry that more closely represents designs with vias to ground on their shorted stubs. Using a classical microstrip configuration, they would need to include a via in the design if introducing any shorts to ground. This is not so easy compared to using a design without vias as they require keying the board to ensure the front and back sides are exactly aligned in addition to drilling and seating the vias if pressed or filling with copper through deposition. As shown in Fig. 2, the shorted stubs are not fully connected to the design region, meaning their inclusion is closer to a capacitive element than it is to a direct short which would introduce more of an inductive element. Additionally, in Fig. 2c, corner regions in the design are connected by an additional patch to ensure connectivity between pixels as was shown in^2^.

**Table 1:** Dimensions used in fabricated design.

Symbol	Quantity	Value (mm)
$\:W$	Design region width	9.6
$\:h$	Design region height	6.4
$\:{b}_{w}$	Board width	26.15
$\:{b}_{h}$	Board height	12.8
$\:g$	Waveguide gap	0.5
$\:s$	Pixel side length	0.2
$\:p$	Pixel corner patch side length	0.1
$\:{k}_{1}$	Length of short 1	0.875
$\:{k}_{1}d$	Distance to short 1	2.982
$\:{k}_{2}$	Length of short 2	0.9315
$\:{k}_{2}d$	Distance to short 2	4.149

To begin the design process, the pixel side length was set to 400 μm. When we subdivide, each pixel side length is cut in half, creating four smaller pixels. This minimizes the amount of time needed to find a candidate as the initial design space is cut down in size by a factor of four. With the pixel density reduced, we can more easily sift through the possible patterns to find a suitable candidate for further refinement. For instance, the design shown in Fig. 2(a) started with 24 × 16 pixels. After a suitable candidate was found, we subdivided the pixels so that each became four new pixels. The final board shown and discussed in this work has 48 × 32 pixels and a pixel side length of 200 μm. To evaluate fitness of the patterns, a figure of merit is defined as the weighted sum of S-parameter values for a two-port network. To define the figure of merit, a goal function is defined as an overall convergence target. This expression serves as the basis for optimization and shown in (1). A goal is set in terms of S-parameters in dB and referred to as the target $\:{T}_{ii}$ whereas the current candidate is $\:{S}_{ii}$. $\:N$ is the number of frequency samples in the simulation, the mean squared error is calculated across frequency, and the sum is taken across the weighted values. This figure of merit is then compared against the best current candidate and iterated on until a better candidate is found.

1 $$\:MSE=\frac{1}{N}\sum\:_{f=1}^{f=N}{\left|{T}_{ii}\right(f)-{S}_{ii}(f\left)\right|}^{2}$$

The mean squared error (MSE) is multiplied by the weights as shown in (2) and this occurs for all elements in the set Ω.

2 $$\:Err=\:\frac{1}{N}\sum\:_{ii\in\:{\Omega\:}}{W}_{ii}\cdot\:MSE,\:\:{\Omega\:}=\left\{11,\:12,\:21,\:22\right\}$$

**Fig. 3:**
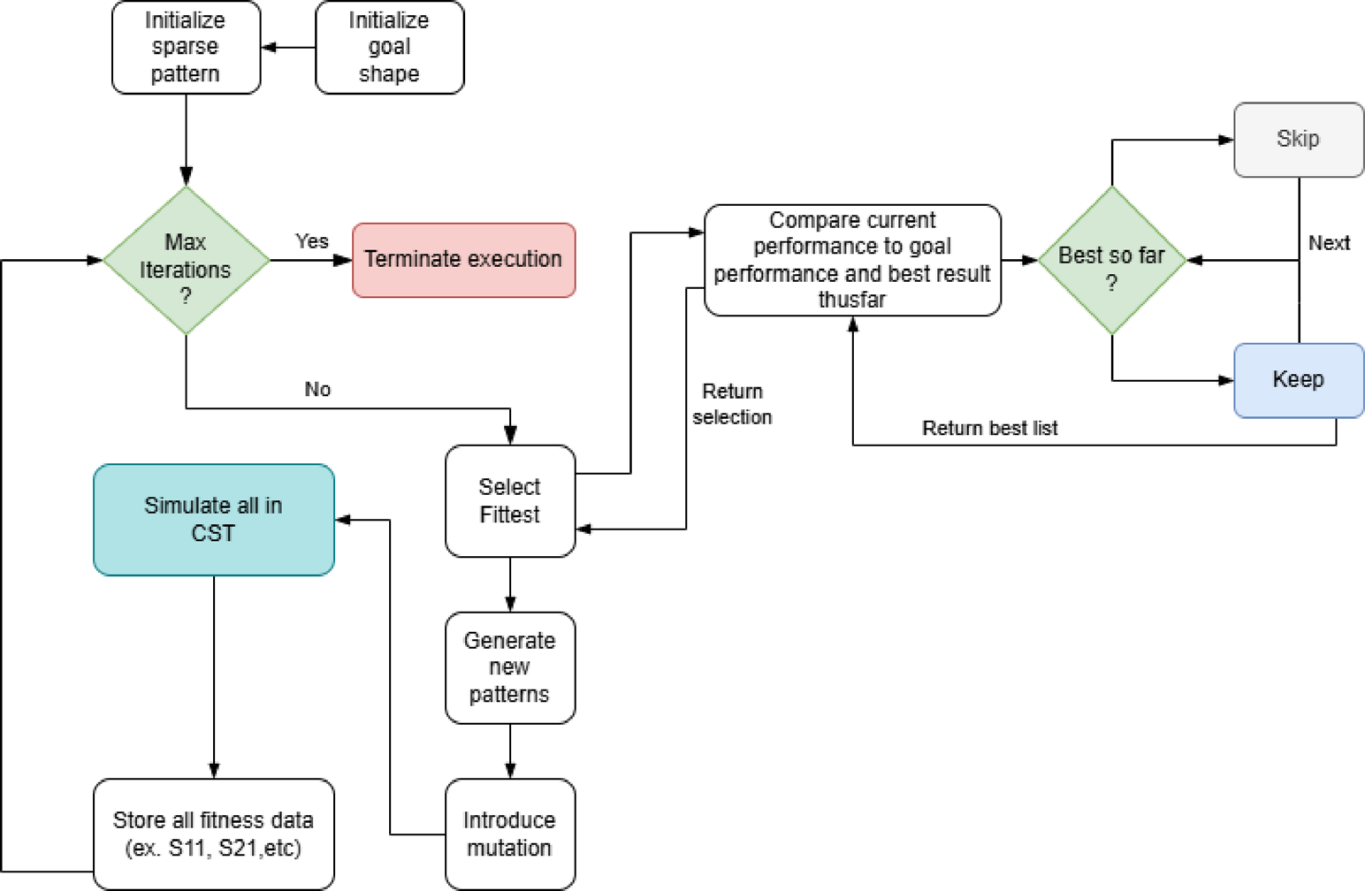
Diagram of the genetic algorithm implementation showing the iterative process from pattern initialization through CST simulation, fitness evaluation, selection, and mutation (settings: 32 population size, 50 max iterations, 60% mutation rate).

This method allows for focusing on particular parameters by setting the weights of the unwanted $\:{S}_{ii}$ to zero. Once a better candidate is found with this metric through the optimization loop shown in Fig. 3, and the maximum iteration stage is complete, each pixel of the initial design region is subdivided into four. This subdivision provides more degrees of freedom for improvement in the design, while beginning with the coarser grid cuts down on the time spent searching for the solution space. The patterned region can continue to be subdivided and iterated upon, but the limitations in fabrication would begin to appear. Starting with a pixel size of 400 μm, we can subdivide the design space twice before we reach the minimum feature size that could be obtained with the manufacturing methods, but a single subdivision is sufficient here. After this refinement, a sensitivity analysis is performed to evaluate the design’s robustness and stability. This analysis helps identify how small variations in the parameters may affect overall performance, ensuring that the design maintains its desired electromagnetic characteristics under realistic operating conditions. Concurrently, adjustments such as introducing shorts are made to further improve the Q factor, effectively deepening the notch in the filter response and narrowing the bandwidth. This step is crucial not only for optimizing the filter’s performance, but it also ensures that the design is practical from both a manufacturing and operational perspective.

**Fig. 4:**
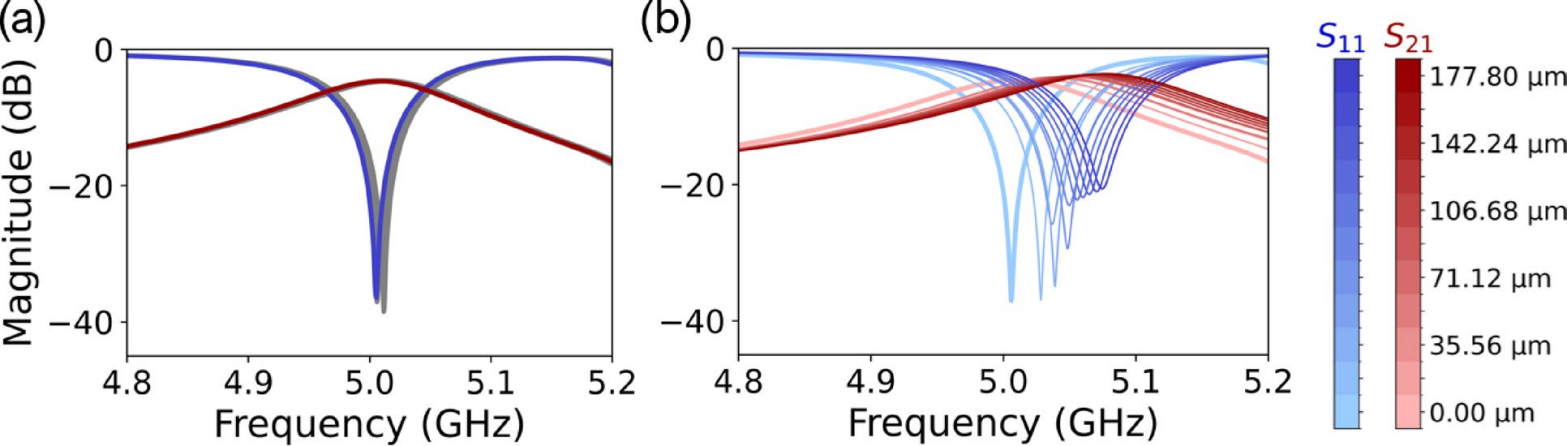
(**a**) Expected filter response for 200 μm pixels with gray traces representing responses when sweeping values of the pixels edge locations, showing the effects of over cutting the pixel boundary. The frequency range was chosen to highlight the target resonance at 5 GHz. (**b**) Sensitivity to depth of dielectric cut. $\:{S}_{11}$ is shown in blue with increasing darkness as cut becomes deeper. $\:{S}_{21}$ shown in red with similar darkening.

Shown in Fig. 4(a) we see in gray that the over cutting of the pixels edges can cause some minor deviations to the peak frequency position, but overall these tend to not shift much. However, the overcutting into the dielectric at various thicknesses, analyzed in Fig. 4(b), shows that the resonance tends to shift in frequency with clear changes in $\:{S}_{11}$ as the depth of the cut into the dielectric increases. In addition to this, we observe that the changes in S-parameters are non-monotonic. For some of these depth cuts, the varying overcut appears to have a monotonic effect while others exhibit deviations from that trend. These effects appear to be difficult to control experimentally. The coupling between pixels in some regions throughout the pattern can have varied effects thus leading to non-trivial shifts of the resulting resonant frequency depending on the depth of the dielectric cut, resulting in deviations from the target. This is something that should be considered when optimizing the fabrication of these devices.

**Fig. 5:**
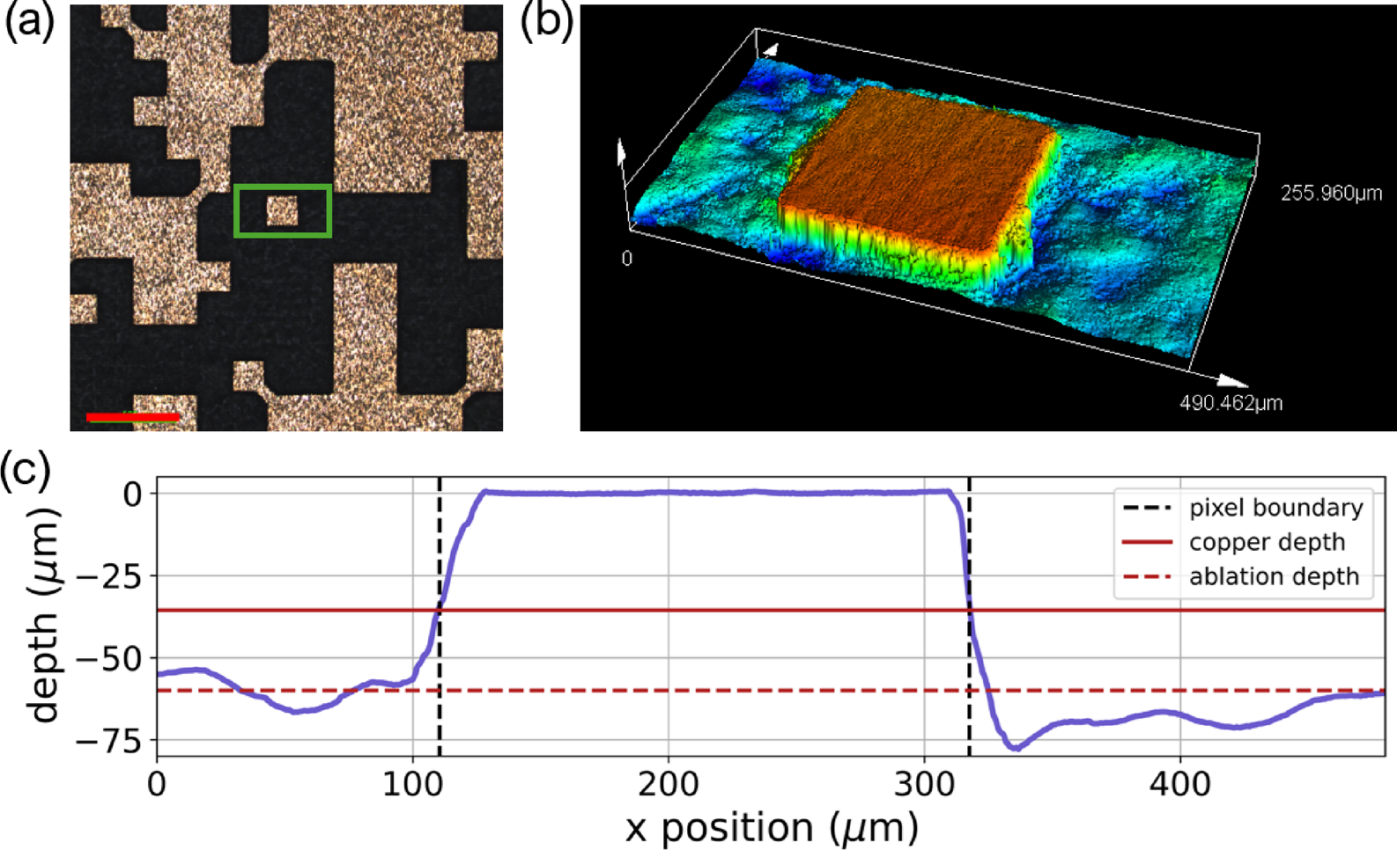
(**a**) Microscope image of the fabricated device with 500 μm scale bar in red. (**b**) Zoom in zone depth measurement. (**c**) Graph showing the depth of the dielectric compared to copper surface.

The fabrication of the designed microwave filter is achieved through laser ablation on the PCB board utilizing a UV laser system of Series 3500 from DPSS Lasers Inc. The microscope image of the fabricated device is shown in Fig. 5(a) with a zoom in zone depth measurement shown in Fig. 5(b). The depth characterization results from Fig. 5(b) measurement is presented in Fig. 5(c). It is evident that there is reasonable agreement between the measured side length $\:s$ and the design specifications. We have an average value of 185 μm across the pixels measured and this represents an approximate error of 7.5%. With further refinement of the laser ablation parameters (power, focus, and scanning speed) and potential post-processing techniques, this fabrication error can be significantly reduced to achieve closer agreement between designed and fabricated dimensions, thereby improving frequency targeting precision.

**Fig. 6:**
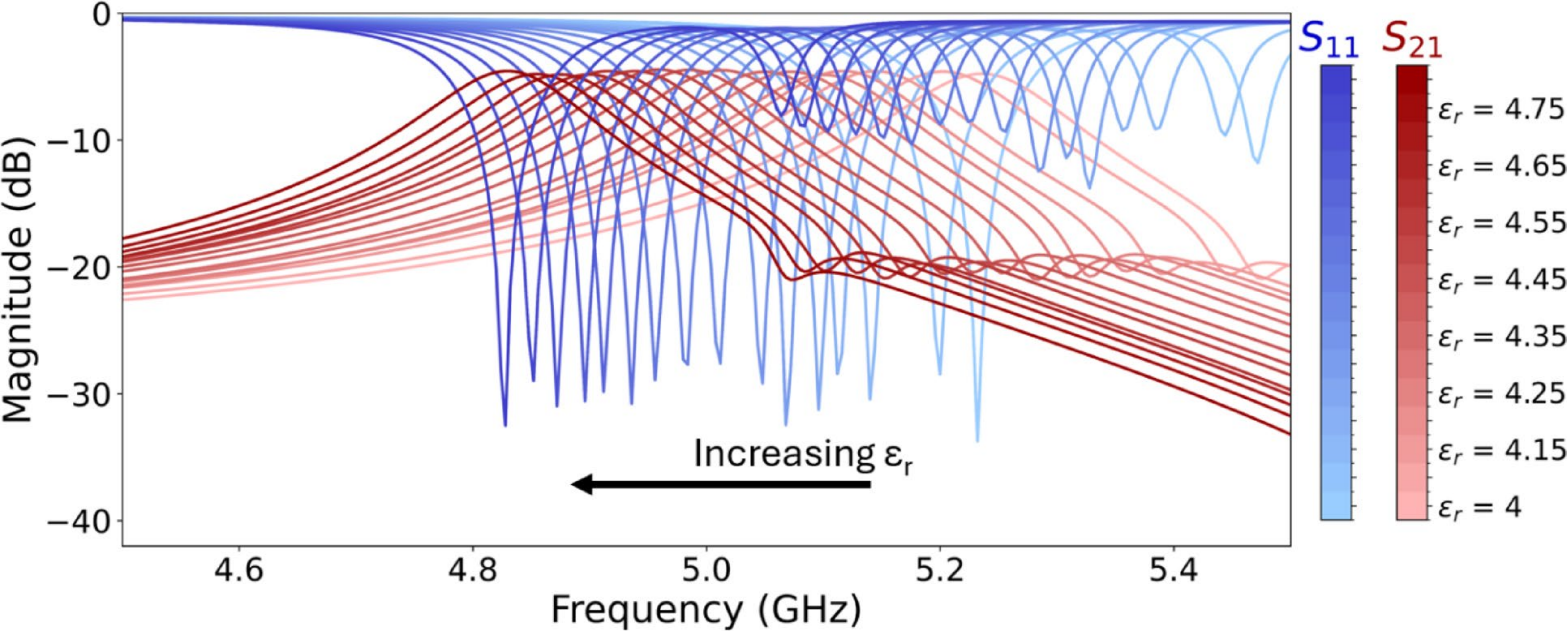
Sensitivity to dielectric constant. $\:{S}_{11}$ shown in blue with increasing darkness as relative permittivity increases. $\:{S}_{21}$ shown in red with similar darkening. The above sweep was taken with the known overcut average and over-etching into the dielectric.

Once we obtained the measurements for the surface of the device, another sweep was performed to see the effects of variation in the substrate dielectric constant using these measured deviations. The sweep shown in Fig. 6 clearly depicts a deviation from the center frequency as the dielectric constant is changed from ± 10% of the expected value of 4.38; with lighter traces representing a lower dielectric constant.

## Results and discussion

While previous works like^18^ have demonstrated stopband-enhanced filters using ring resonators and^19^ explored short-circuited coupling elements, this approach uniquely combines pixelated metasurfaces with integrated shorted stubs to achieve significant notch depth improvement (~ 35 dB) and bandwidth enhancement (~ 10%) without requiring additional circuit layers or via structures—offering a more compact and manufacturing-friendly alternative.

A traditional design that was analyzed when considering filter compactness was that of a 3rd order Chebyshev interdigitated filter. For a bandpass filter with interdigital topology, we would require a combined gap and conductor width of approximately 25 mm and a conductor length from open end to shorted via of 8 mm. This results in a design region of 200 mm^2^. When comparing to our proposed design, which consists of a design region of 9.6 mm by 6.4 mm thus an area of 61.5 mm^2^, the proposed filter is less than 50% the size of the Chebyshev filter. Overall, the full board design leads to a significant reduction in overall area while having the added benefit of being a single-layer design without the need for vias.

Tunability of the passband response can be attainable through multiple approaches. First by definition of the goal function. Should a different target frequency be desired, one would include this in the target goal to have the iterative inverse design loop prioritize any frequency. This enables the design of multiple passive filters with pre-selected frequency response. In addition to the target definition, we have seen through the results of the sensitivity analysis that the cut-depth, pixel overcut, and permittivity could be included as additional degrees of freedom to assist the loop in converging to the desired filter geometry for a desired resonance. Controlling these parameters can enable the fabrication of a filter at different target frequency without the need to re-optimize the geometry. Furthermore, active dynamic tuning is possible by employing reconfigurable materials, such as liquid crystals and phase change materials.

**Fig. 7:**
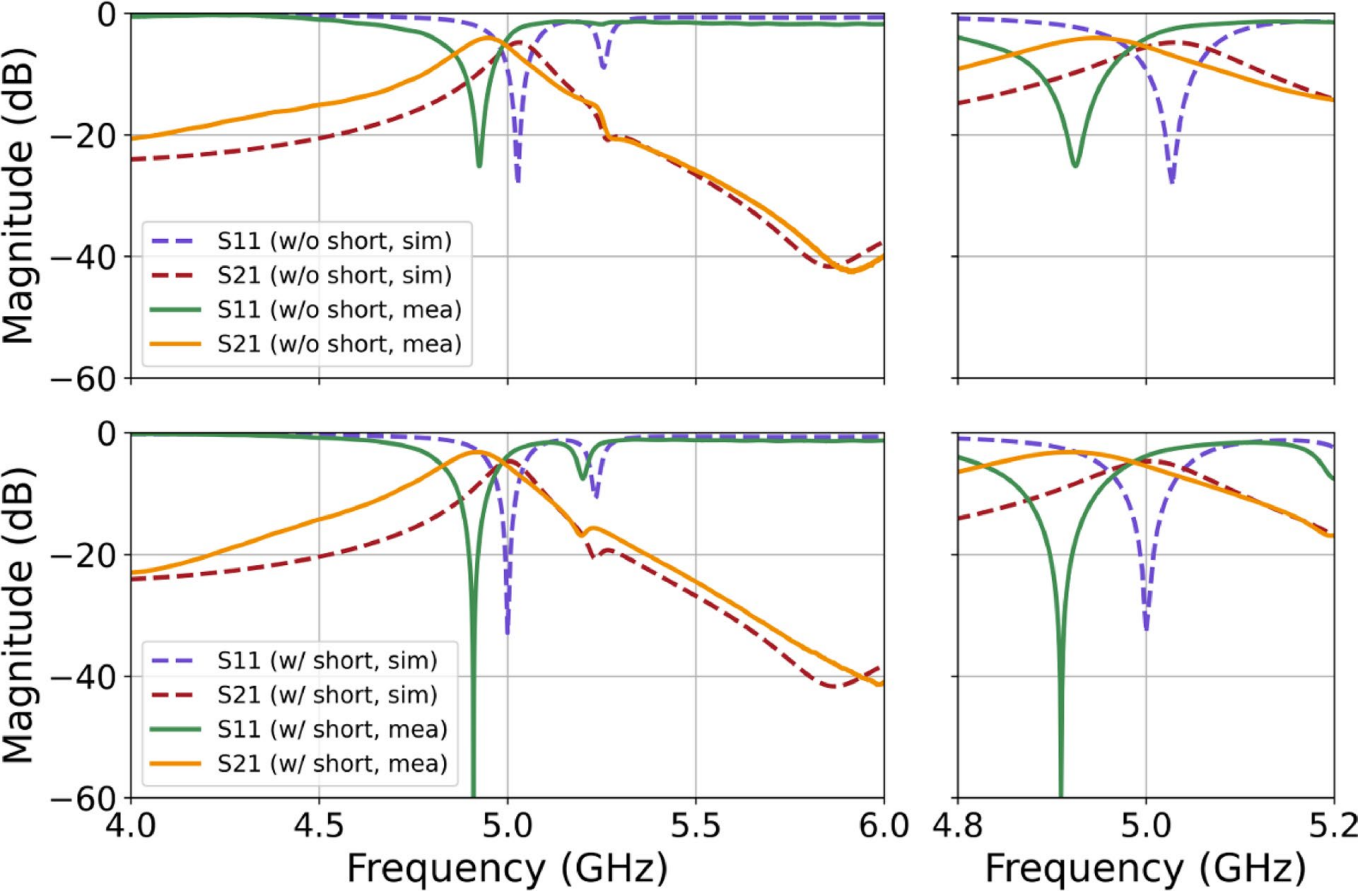
Frequency response of the designed bandpass microwave filter without shorts (left top figure) and with shorts (left bottom figure), highlighting the performance improvements. Closeup of target region without shorts (right top figure) and with shorts (bottom right figure).

Shown in Fig. 7 is the simulated filter response and the captured results from the fabricated filter with and without shorts. The overall response is strong at the target of 5 GHz in the simulation; however, we see a frequency shift in the measured filter response. Given the sensitivity analysis shown in the previous section, it is likely that the differences seen in the position of the resonant frequency could be attributed to a combination of factors with the largest contributor being the dielectric constant of the substrate in addition to the pixel overcut and deeper cut-depth of the dielectric. Though we do not have perfect agreement in $\:{S}_{11}$ in the measured filter, we have an adequate match in the shape of the response compared to the simulation. The captured result shown in the bottom graph of Fig. 7 shows the response of the fabricated filter with the included shorts. Here too we see the same discrepancy in the target band and the measured center frequency for the filter is shifted by 160 MHz. This discrepancy can be understood on the basis of fabrication byproducts, particularly the value of the dielectric constant. Though the center is not where we expect, the shape of the resonance and frequency response agrees with simulation, and we observe an improvement in the depth of the notch when shorted, as well as a shift closer to the target frequency. The overall idea that including shorts in the design would provide a deeper notch has been demonstrated. The filter without shorting has a measured minimum of −26 dB, while the shorted filter obtained a minimum of −61 dB, yielding a ~ 35 dB improvement in notch depth. Measuring the difference between the two measured results shows an improvement in bandwidth for the shorted filter of ~ 10%. The target of both filters is 200 MHz with the shorted filter coming in at 248 MHz compared to the non-shorted filter of 265 MHz.

## Conclusion

It is critical to expand the existing design framework by combining multiple optimization strategies and configurations to overcome the inherent limitations of single-layer microstrip pixel-based approaches. The approach presented in this work offers considerable practical advantages compared to conventional designs: integration of shorted stubs with pixelated metasurfaces eliminates the need for additional layers, vias, or complex fabrication steps while still achieving superior performance. This effectively reduces manufacturing complexity and cost while maintaining desired electromagnetic characteristics found in designs with additional complexity, demonstrating that performance improvements can be achieved without sacrificing practicality. Further refinement is required for laser ablation designs to mitigate the swings in center frequency and resonance strength that appear from deviations in cutting depth and pixel size. This refinement would allow for the designs in this work to perform closer to the desired and simulated behavior. Inverse-design methodologies can serve as powerful tools for systematically refining structures to meet target objectives. Building on these principles, notch filters with integrated stubs offer a promising route for enhancing performance. Moreover, subdividing pixel-based patterns once initial candidates are identified provides finer control over the final design. Looking ahead, this proposed methodology can be adapted to various other components, including high-pass and low-pass filters, frequency splitters, or even active devices with tunable frequency responses - potentially unlocking new frontiers in advanced microwave filter design and signal management.

## Data Availability

Data underlying the results presented in this paper are not publicly available at this time but may be obtained from the corresponding authors upon reasonable request.
